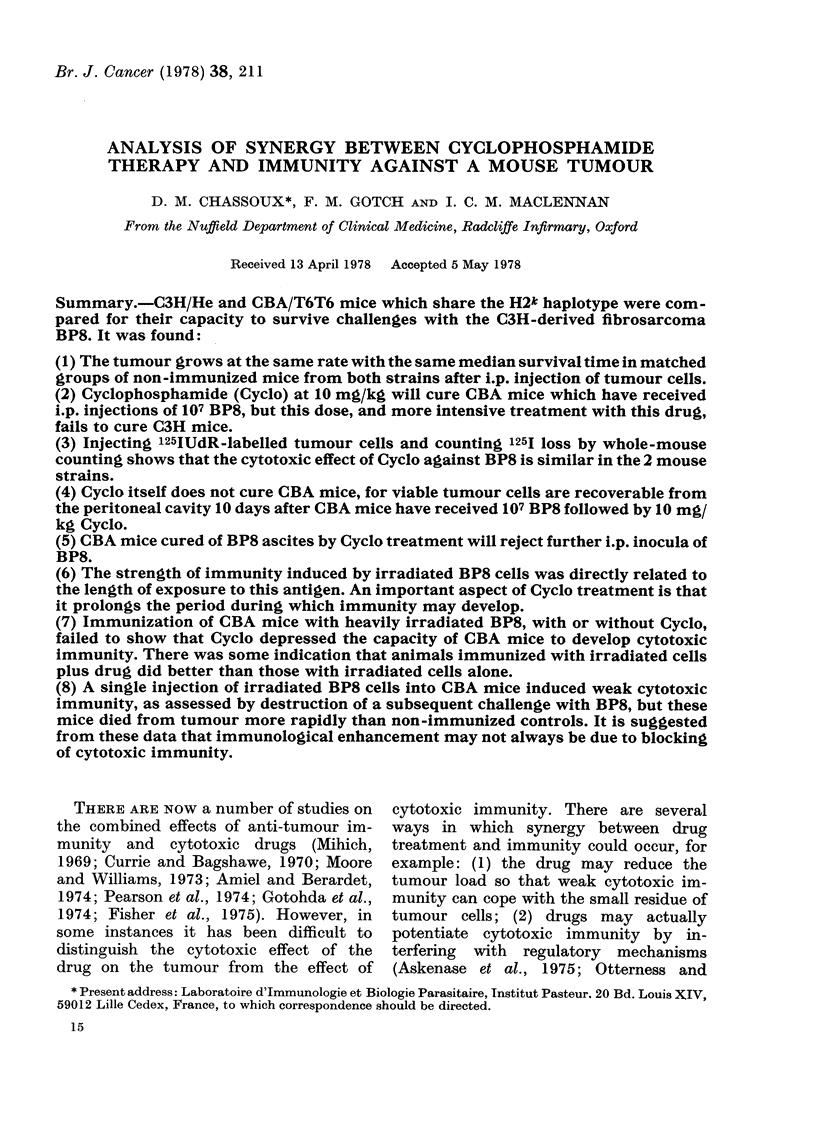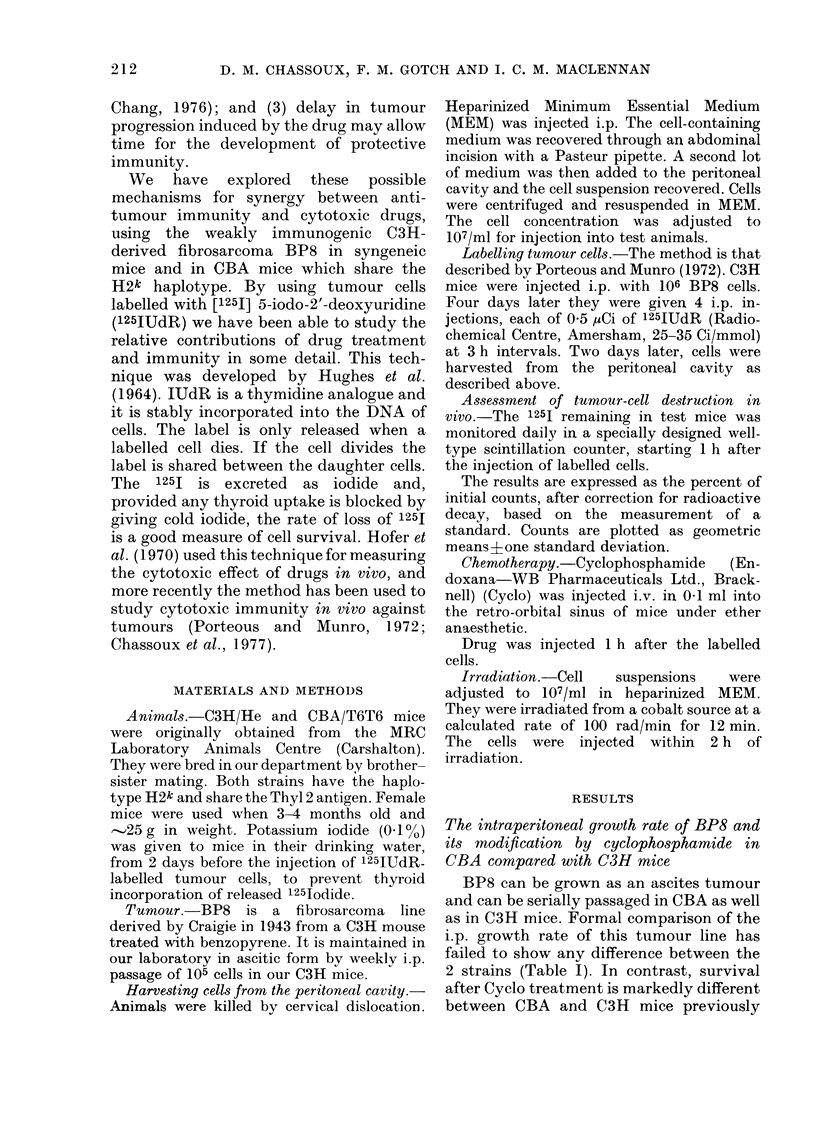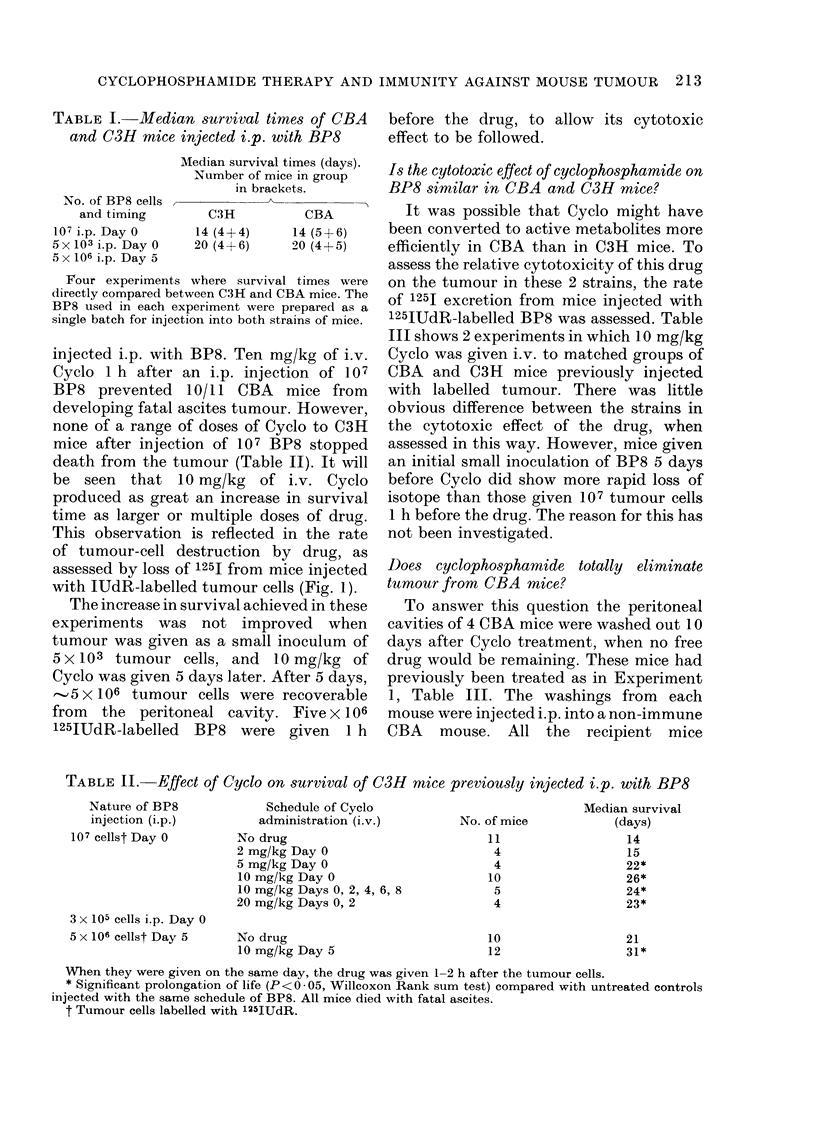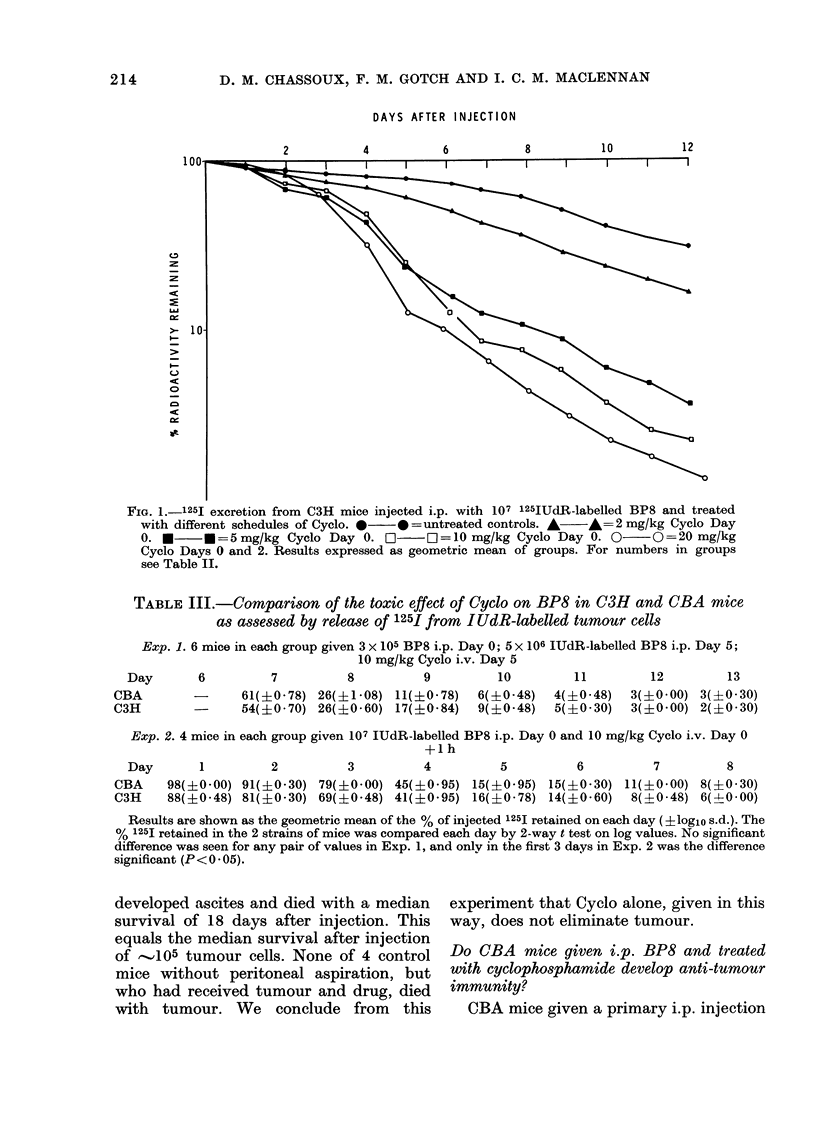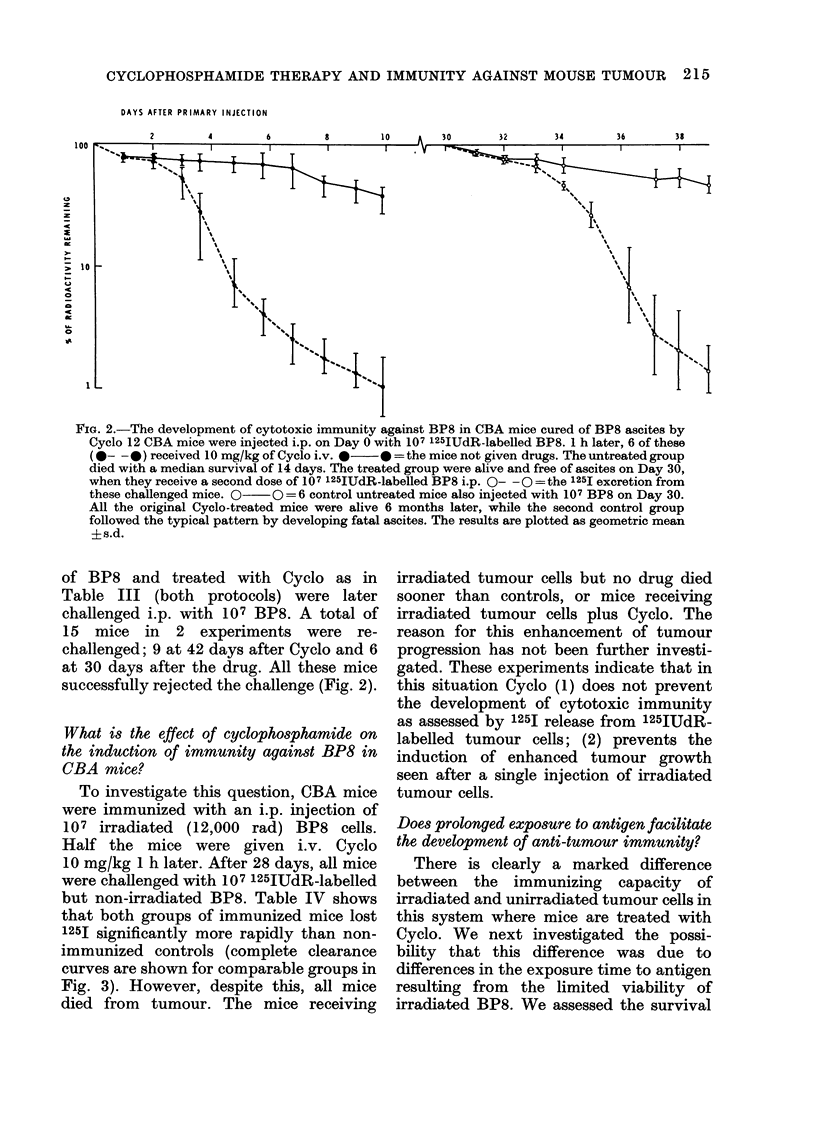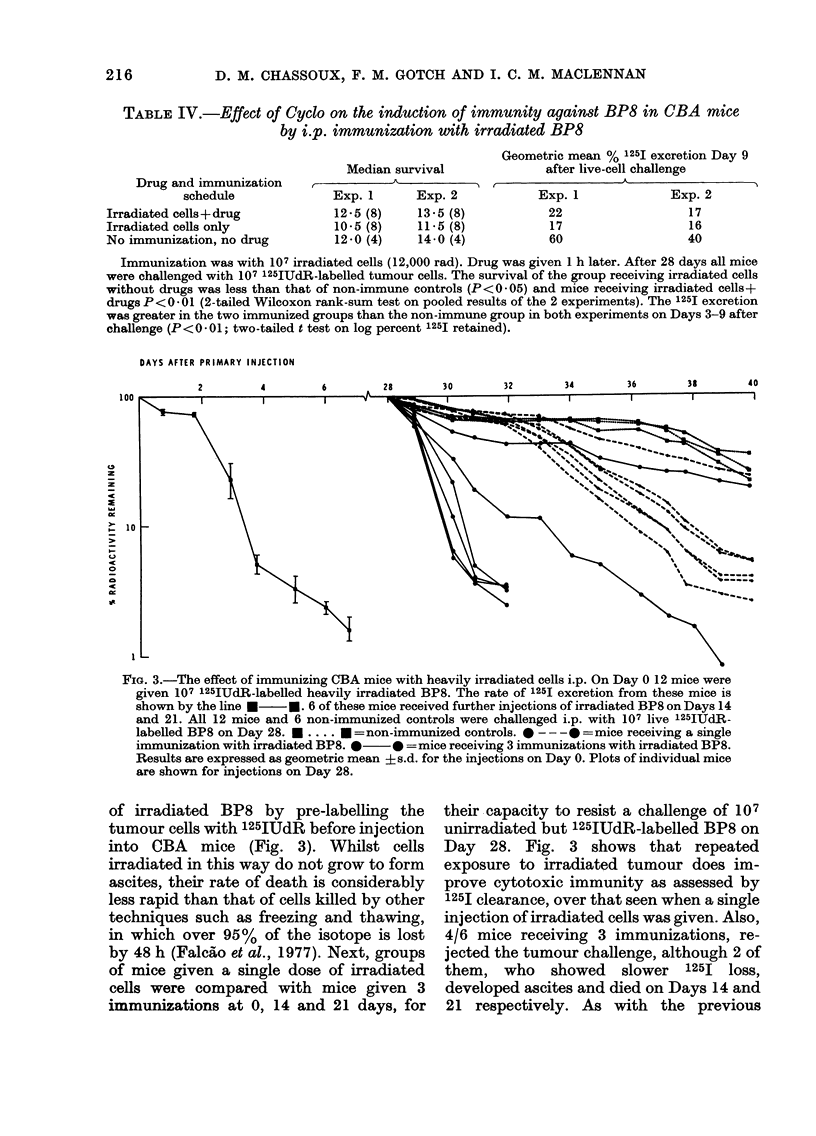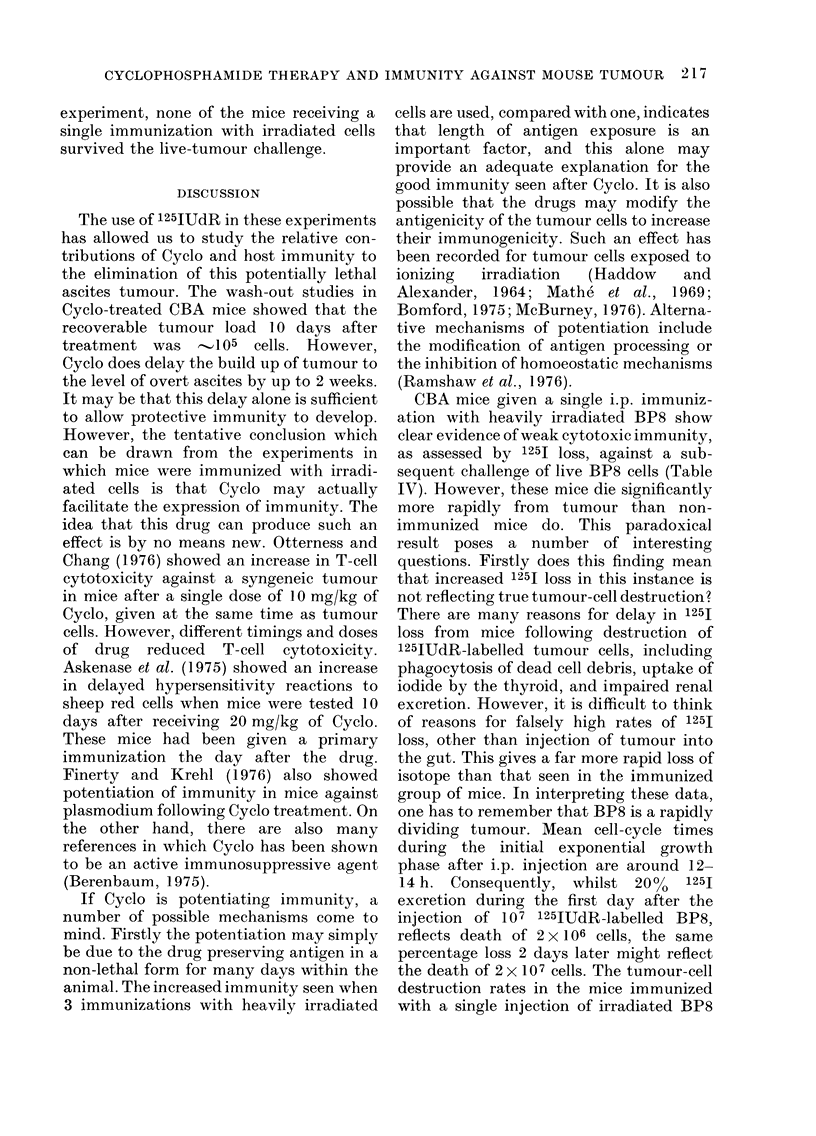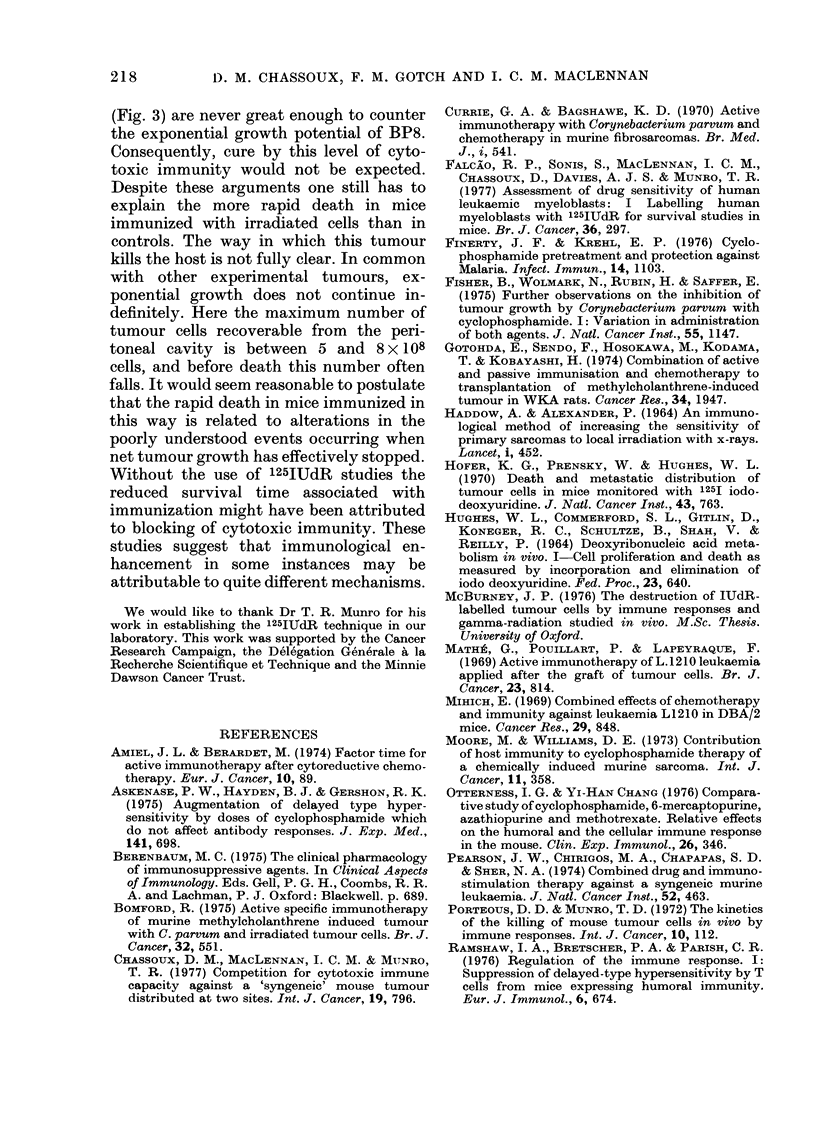# Analysis of synergy between cyclophosphamide Therapy and Immunity Against a Mouse Tumour

**DOI:** 10.1038/bjc.1978.190

**Published:** 1978-08

**Authors:** D. M. Chassoux, F. M. Gotch, I. C. M. MacLennan

## Abstract

C3H/He and CBA/T6T6 mice which share the H2^*k*^ haplotype were compared for their capacity to survive challenges with the C3H-derived fibrosarcoma BP8. It was found:

(1) The tumour grows at the same rate with the same median survival time in matched groups of non-immunized mice from both strains after i.p. injection of tumour cells.

(2) Cyclophosphamide (Cyclo) at 10 mg/kg will cure CBA mice which have received i.p. injections of 10^7^ BP8, but this dose, and more intensive treatment with this drug, fails to cure C3H mice.

(3) Injecting ^125^IUdR-labelled tumour cells and counting ^125^I loss by whole-mouse counting shows that the cytotoxic effect of Cyclo against BP8 is similar in the 2 mouse strains.

(4) Cyclo itself does not cure CBA mice, for viable tumour cells are recoverable from the peritoneal cavity 10 days after CBA mice have received 10^7^ BP8 followed by 10 mg/kg Cyclo.

(5) CBA mice cured of BP8 ascites by Cyclo treatment will reject further i.p. inocula of BP8.

(6) The strength of immunity induced by irradiated BP8 cells was directly related to the length of exposure to this antigen. An important aspect of Cyclo treatment is that it prolongs the period during which immunity may develop.

(7) Immunization of CBA mice with heavily irradiated BP8, with or without Cyclo, failed to show that Cyclo depressed the capacity of CBA mice to develop cytotoxic immunity. There was some indication that animals immunized with irradiated cells plus drug did better than those with irradiated cells alone.

(8) A single injection of irradiated BP8 cells into CBA mice induced weak cytotoxic immunity, as assessed by destruction of a subsequent challenge with BP8, but these mice died from tumour more rapidly than non-immunized controls. It is suggested from these data that immunological enhancement may not always be due to blocking of cytotoxic immunity.


*Br. J. Cancer* (1978) **38**, 211

### ANALYSIS OF SYNERGY BETWEEN CYCLOPHOSPHAMIDE THERAPY AND IMMUNITY AGAINST A MOUSE TUMOUR

D. M. CHASSOUX*, F. M. GOTCH AND I. C. M. MACLENNAN

*From the Nuffield Department of Clinical Medicine, Radcliffe Infirmary, Oxford*

Received 13 April 1978 Accepted 5 May 1978

**Summary.—C3H/He and CBA/T6T6 mice which share the H2$^k$ haplotype were compared for their capacity to survive challenges with the C3H-derived fibrosarcoma BP8. It was found:**

**(1) The tumour grows at the same rate with the same median survival time in matched groups of non-immunized mice from both strains after i.p. injection of tumour cells.**
**(2) Cyclophosphamide (Cyclo) at 10 mg/kg will cure CBA mice which have received i.p. injections of $10^7$ BP8, but this dose, and more intensive treatment with this drug, fails to cure C3H mice.**
**(3) Injecting $^{125}$IUdR-labelled tumour cells and counting $^{125}$I loss by whole-mouse counting shows that the cytotoxic effect of Cyclo against BP8 is similar in the 2 mouse strains.**
**(4) Cyclo itself does not cure CBA mice, for viable tumour cells are recoverable from the peritoneal cavity 10 days after CBA mice have received $10^7$ BP8 followed by 10 mg/kg Cyclo.**
**(5) CBA mice cured of BP8 ascites by Cyclo treatment will reject further i.p. inocula of BP8.**
**(6) The strength of immunity induced by irradiated BP8 cells was directly related to the length of exposure to this antigen. An important aspect of Cyclo treatment is that it prolongs the period during which immunity may develop.**
**(7) Immunization of CBA mice with heavily irradiated BP8, with or without Cyclo, failed to show that Cyclo depressed the capacity of CBA mice to develop cytotoxic immunity. There was some indication that animals immunized with irradiated cells plus drug did better than those with irradiated cells alone.**
**(8) A single injection of irradiated BP8 cells into CBA mice induced weak cytotoxic immunity, as assessed by destruction of a subsequent challenge with BP8, but these mice died from tumour more rapidly than non-immunized controls. It is suggested from these data that immunological enhancement may not always be due to blocking of cytotoxic immunity.**

THERE ARE NOW a number of studies on the combined effects of anti-tumour immunity and cytotoxic drugs (Mihich, 1969; Currie and Bagshawe, 1970; Moore and Williams, 1973; Amiel and Berardet, 1974; Pearson *et al.*, 1974; Gotohda *et al.*, 1974; Fisher *et al.*, 1975). However, in some instances it has been difficult to distinguish the cytotoxic effect of the drug on the tumour from the effect of cytotoxic immunity. There are several ways in which synergy between drug treatment and immunity could occur, for example: (1) the drug may reduce the tumour load so that weak cytotoxic immunity can cope with the small residue of tumour cells; (2) drugs may actually potentiate cytotoxic immunity by interfering with regulatory mechanisms (Askenase *et al.*, 1975; Otterness and

* Present address: Laboratoire d'Immunologie et Biologie Parasitaire, Institut Pasteur. 20 Bd. Louis XIV, 59012 Lille Cedex, France, to which correspondence should be directed.

Chang, 1976); and (3) delay in tumour progression induced by the drug may allow time for the development of protective immunity.

We have explored these possible mechanisms for synergy between anti-tumour immunity and cytotoxic drugs, using the weakly immunogenic C3H-derived fibrosarcoma BP8 in syngeneic mice and in CBA mice which share the $H2^k$ haplotype. By using tumour cells labelled with [$^{125}$I] 5-iodo-2′-deoxyuridine ($^{125}$IUdR) we have been able to study the relative contributions of drug treatment and immunity in some detail. This technique was developed by Hughes *et al.* (1964). IUdR is a thymidine analogue and it is stably incorporated into the DNA of cells. The label is only released when a labelled cell dies. If the cell divides the label is shared between the daughter cells. The $^{125}$I is excreted as iodide and, provided any thyroid uptake is blocked by giving cold iodide, the rate of loss of $^{125}$I is a good measure of cell survival. Hofer *et al.* (1970) used this technique for measuring the cytotoxic effect of drugs *in vivo*, and more recently the method has been used to study cytotoxic immunity *in vivo* against tumours (Porteous and Munro, 1972; Chassoux *et al.*, 1977).

### MATERIALS AND METHODS

*Animals.*—C3H/He and CBA/T6T6 mice were originally obtained from the MRC Laboratory Animals Centre (Carshalton). They were bred in our department by brother–sister mating. Both strains have the haplotype $H2^k$ and share the Thyl 2 antigen. Female mice were used when 3–4 months old and ~25 g in weight. Potassium iodide (0·1%) was given to mice in their drinking water, from 2 days before the injection of $^{125}$IUdR-labelled tumour cells, to prevent thyroid incorporation of released $^{125}$Iodide.

*Tumour.*—BP8 is a fibrosarcoma line derived by Craigie in 1943 from a C3H mouse treated with benzopyrene. It is maintained in our laboratory in ascitic form by weekly i.p. passage of $10^5$ cells in our C3H mice.

*Harvesting cells from the peritoneal cavity.*—Animals were killed by cervical dislocation. Heparinized Minimum Essential Medium (MEM) was injected i.p. The cell-containing medium was recovered through an abdominal incision with a Pasteur pipette. A second lot of medium was then added to the peritoneal cavity and the cell suspension recovered. Cells were centrifuged and resuspended in MEM. The cell concentration was adjusted to $10^7$/ml for injection into test animals.

*Labelling tumour cells.*—The method is that described by Porteous and Munro (1972). C3H mice were injected i.p. with $10^6$ BP8 cells. Four days later they were given 4 i.p. injections, each of 0·5 $\mu$Ci of $^{125}$IUdR (Radiochemical Centre, Amersham, 25–35 Ci/mmol) at 3 h intervals. Two days later, cells were harvested from the peritoneal cavity as described above.

*Assessment of tumour-cell destruction in vivo.*—The $^{125}$I remaining in test mice was monitored daily in a specially designed well-type scintillation counter, starting 1 h after the injection of labelled cells.

The results are expressed as the percent of initial counts, after correction for radioactive decay, based on the measurement of a standard. Counts are plotted as geometric means $\pm$ one standard deviation.

*Chemotherapy.*—Cyclophosphamide (Endoxana—WB Pharmaceuticals Ltd., Bracknell) (Cyclo) was injected i.v. in 0·1 ml into the retro-orbital sinus of mice under ether anaesthetic.

Drug was injected 1 h after the labelled cells.

*Irradiation.*—Cell suspensions were adjusted to $10^7$/ml in heparinized MEM. They were irradiated from a cobalt source at a calculated rate of 100 rad/min for 12 min. The cells were injected within 2 h of irradiation.

### RESULTS

### *The intraperitoneal growth rate of BP8 and its modification by cyclophosphamide in CBA compared with C3H mice*

BP8 can be grown as an ascites tumour and can be serially passaged in CBA as well as in C3H mice. Formal comparison of the i.p. growth rate of this tumour line has failed to show any difference between the 2 strains (Table I). In contrast, survival after Cyclo treatment is markedly different between CBA and C3H mice previously

TABLE I.—*Median survival times of CBA and C3H mice injected i.p. with BP8*

| No. of BP8 cells and timing | Median survival times (days). Number of mice in group in brackets. | |
|---|---|---|
| | C3H | CBA |
| $10^7$ i.p. Day 0 | 14 (4+4) | 14 (5+6) |
| $5\times10^3$ i.p. Day 0 $5\times10^6$ i.p. Day 5 | 20 (4+6) | 20 (4+5) |

Four experiments where survival times were directly compared between C3H and CBA mice. The BP8 used in each experiment were prepared as a single batch for injection into both strains of mice.

injected i.p. with BP8. Ten mg/kg of i.v. Cyclo 1 h after an i.p. injection of $10^7$ BP8 prevented 10/11 CBA mice from developing fatal ascites tumour. However, none of a range of doses of Cyclo to C3H mice after injection of $10^7$ BP8 stopped death from the tumour (Table II). It will be seen that 10 mg/kg of i.v. Cyclo produced as great an increase in survival time as larger or multiple doses of drug. This observation is reflected in the rate of tumour-cell destruction by drug, as assessed by loss of $^{125}$I from mice injected with IUdR-labelled tumour cells (Fig. 1).

The increase in survival achieved in these experiments was not improved when tumour was given as a small inoculum of $5\times10^3$ tumour cells, and 10 mg/kg of Cyclo was given 5 days later. After 5 days, $\sim5\times10^6$ tumour cells were recoverable from the peritoneal cavity. Five $\times10^6$ $^{125}$IUdR-labelled BP8 were given 1 h before the drug, to allow its cytotoxic effect to be followed.

*Is the cytotoxic effect of cyclophosphamide on BP8 similar in CBA and C3H mice?*

It was possible that Cyclo might have been converted to active metabolites more efficiently in CBA than in C3H mice. To assess the relative cytotoxicity of this drug on the tumour in these 2 strains, the rate of $^{125}$I excretion from mice injected with $^{125}$IUdR-labelled BP8 was assessed. Table III shows 2 experiments in which 10 mg/kg Cyclo was given i.v. to matched groups of CBA and C3H mice previously injected with labelled tumour. There was little obvious difference between the strains in the cytotoxic effect of the drug, when assessed in this way. However, mice given an initial small inoculation of BP8 5 days before Cyclo did show more rapid loss of isotope than those given $10^7$ tumour cells 1 h before the drug. The reason for this has not been investigated.

*Does cyclophosphamide totally eliminate tumour from CBA mice?*

To answer this question the peritoneal cavities of 4 CBA mice were washed out 10 days after Cyclo treatment, when no free drug would be remaining. These mice had previously been treated as in Experiment 1, Table III. The washings from each mouse were injected i.p. into a non-immune CBA mouse. All the recipient mice

TABLE II.—*Effect of Cyclo on survival of C3H mice previously injected i.p. with BP8*

| Nature of BP8 injection (i.p.) | Schedule of Cyclo administration (i.v.) | No. of mice | Median survival (days) |
|---|---|---|---|
| $10^7$ cells† Day 0 | No drug | 11 | 14 |
| | 2 mg/kg Day 0 | 4 | 15 |
| | 5 mg/kg Day 0 | 4 | 22* |
| | 10 mg/kg Day 0 | 10 | 26* |
| | 10 mg/kg Days 0, 2, 4, 6, 8 | 5 | 24* |
| | 20 mg/kg Days 0, 2 | 4 | 23* |
| $3\times10^5$ cells i.p. Day 0 | | | |
| $5\times10^6$ cells† Day 5 | No drug | 10 | 21 |
| | 10 mg/kg Day 5 | 12 | 31* |

When they were given on the same day, the drug was given 1–2 h after the tumour cells.

* Significant prolongation of life ($P<0\cdot05$, Willcoxon Rank sum test) compared with untreated controls injected with the same schedule of BP8. All mice died with fatal ascites.

† Tumour cells labelled with $^{125}$IUdR.

FIG. 1.—$^{125}$I excretion from C3H mice injected i.p. with $10^7$ $^{125}$IUdR-labelled BP8 and treated with different schedules of Cyclo. ●——● = untreated controls. ▲——▲ = 2 mg/kg Cyclo Day 0. ■——■ = 5 mg/kg Cyclo Day 0. □——□ = 10 mg/kg Cyclo Day 0. ○——○ = 20 mg/kg Cyclo Days 0 and 2. Results expressed as geometric mean of groups. For numbers in groups see Table II.

TABLE III.—*Comparison of the toxic effect of Cyclo on BP8 in C3H and CBA mice as assessed by release of $^{125}$I from IUdR-labelled tumour cells*

*Exp. 1.* 6 mice in each group given $3 \times 10^5$ BP8 i.p. Day 0; $5 \times 10^6$ IUdR-labelled BP8 i.p. Day 5; 10 mg/kg Cyclo i.v. Day 5

| Day | 6 | 7 | 8 | 9 | 10 | 11 | 12 | 13 |
|---|---|---|---|---|---|---|---|---|
| CBA | — | 61(±0·78) | 26(±1·08) | 11(±0·78) | 6(±0·48) | 4(±0·48) | 3(±0·00) | 3(±0·30) |
| C3H | — | 54(±0·70) | 26(±0·60) | 17(±0·84) | 9(±0·48) | 5(±0·30) | 3(±0·00) | 2(±0·30) |

*Exp. 2.* 4 mice in each group given $10^7$ IUdR-labelled BP8 i.p. Day 0 and 10 mg/kg Cyclo i.v. Day 0 + 1 h

| Day | 1 | 2 | 3 | 4 | 5 | 6 | 7 | 8 |
|---|---|---|---|---|---|---|---|---|
| CBA | 98(±0·00) | 91(±0·30) | 79(±0·00) | 45(±0·95) | 15(±0·95) | 15(±0·30) | 11(±0·00) | 8(±0·30) |
| C3H | 88(±0·48) | 81(±0·30) | 69(±0·48) | 41(±0·95) | 16(±0·78) | 14(±0·60) | 8(±0·48) | 6(±0·00) |

Results are shown as the geometric mean of the % of injected $^{125}$I retained on each day ($\pm \log_{10}$ s.d.). The % $^{125}$I retained in the 2 strains of mice was compared each day by 2-way *t* test on log values. No significant difference was seen for any pair of values in Exp. 1, and only in the first 3 days in Exp. 2 was the difference significant ($P < 0 \cdot 05$).

developed ascites and died with a median survival of 18 days after injection. This equals the median survival after injection of $\sim 10^5$ tumour cells. None of 4 control mice without peritoneal aspiration, but who had received tumour and drug, died with tumour. We conclude from this experiment that Cyclo alone, given in this way, does not eliminate tumour.

*Do CBA mice given i.p. BP8 and treated with cyclophosphamide develop anti-tumour immunity?*

CBA mice given a primary i.p. injection

FIG. 2.—The development of cytotoxic immunity against BP8 in CBA mice cured of BP8 ascites by Cyclo 12 CBA mice were injected i.p. on Day 0 with $10^7$ $^{125}$IUdR-labelled BP8. 1 h later, 6 of these (●- -●) received 10 mg/kg of Cyclo i.v. ●——● = the mice not given drugs. The untreated group died with a median survival of 14 days. The treated group were alive and free of ascites on Day 30, when they receive a second dose of $10^7$ $^{125}$IUdR-labelled BP8 i.p. ○- -○ = the $^{125}$I excretion from these challenged mice. ○——○ = 6 control untreated mice also injected with $10^7$ BP8 on Day 30. All the original Cyclo-treated mice were alive 6 months later, while the second control group followed the typical pattern by developing fatal ascites. The results are plotted as geometric mean ±s.d.

of BP8 and treated with Cyclo as in Table III (both protocols) were later challenged i.p. with $10^7$ BP8. A total of 15 mice in 2 experiments were re-challenged; 9 at 42 days after Cyclo and 6 at 30 days after the drug. All these mice successfully rejected the challenge (Fig. 2).

*What is the effect of cyclophosphamide on the induction of immunity against BP8 in CBA mice?*

To investigate this question, CBA mice were immunized with an i.p. injection of $10^7$ irradiated (12,000 rad) BP8 cells. Half the mice were given i.v. Cyclo 10 mg/kg 1 h later. After 28 days, all mice were challenged with $10^7$ $^{125}$IUdR-labelled but non-irradiated BP8. Table IV shows that both groups of immunized mice lost $^{125}$I significantly more rapidly than non-immunized controls (complete clearance curves are shown for comparable groups in Fig. 3). However, despite this, all mice died from tumour. The mice receiving irradiated tumour cells but no drug died sooner than controls, or mice receiving irradiated tumour cells plus Cyclo. The reason for this enhancement of tumour progression has not been further investigated. These experiments indicate that in this situation Cyclo (1) does not prevent the development of cytotoxic immunity as assessed by $^{125}$I release from $^{125}$IUdR-labelled tumour cells; (2) prevents the induction of enhanced tumour growth seen after a single injection of irradiated tumour cells.

*Does prolonged exposure to antigen facilitate the development of anti-tumour immunity?*

There is clearly a marked difference between the immunizing capacity of irradiated and unirradiated tumour cells in this system where mice are treated with Cyclo. We next investigated the possibility that this difference was due to differences in the exposure time to antigen resulting from the limited viability of irradiated BP8. We assessed the survival

TABLE IV.—*Effect of Cyclo on the induction of immunity against BP8 in CBA mice by i.p. immunization with irradiated BP8*

| Drug and immunization schedule | Median survival | | Geometric mean % $^{125}$I excretion Day 9 after live-cell challenge | |
|---|---|---|---|---|
| | Exp. 1 | Exp. 2 | Exp. 1 | Exp. 2 |
| Irradiated cells + drug | 12·5 (8) | 13·5 (8) | 22 | 17 |
| Irradiated cells only | 10·5 (8) | 11·5 (8) | 17 | 16 |
| No immunization, no drug | 12·0 (4) | 14·0 (4) | 60 | 40 |

Immunization was with $10^7$ irradiated cells (12,000 rad). Drug was given 1 h later. After 28 days all mice were challenged with $10^7$ $^{125}$IUdR-labelled tumour cells. The survival of the group receiving irradiated cells without drugs was less than that of non-immune controls ($P<0·05$) and mice receiving irradiated cells+drugs $P<0·01$ (2-tailed Wilcoxon rank-sum test on pooled results of the 2 experiments). The $^{125}$I excretion was greater in the two immunized groups than the non-immune group in both experiments on Days 3–9 after challenge ($P<0·01$; two-tailed $t$ test on log percent $^{125}$I retained).

FIG. 3.—The effect of immunizing CBA mice with heavily irradiated cells i.p. On Day 0 12 mice were given $10^7$ $^{125}$IUdR-labelled heavily irradiated BP8. The rate of $^{125}$I excretion from these mice is shown by the line ■——■. 6 of these mice received further injections of irradiated BP8 on Days 14 and 21. All 12 mice and 6 non-immunized controls were challenged i.p. with $10^7$ live $^{125}$IUdR-labelled BP8 on Day 28. ■ . . . . ■ = non-immunized controls. ● – – – ● = mice receiving a single immunization with irradiated BP8. ●——● = mice receiving 3 immunizations with irradiated BP8. Results are expressed as geometric mean ±s.d. for the injections on Day 0. Plots of individual mice are shown for injections on Day 28.

of irradiated BP8 by pre-labelling the tumour cells with $^{125}$IUdR before injection into CBA mice (Fig. 3). Whilst cells irradiated in this way do not grow to form ascites, their rate of death is considerably less rapid than that of cells killed by other techniques such as freezing and thawing, in which over 95% of the isotope is lost by 48 h (Falcão *et al.*, 1977). Next, groups of mice given a single dose of irradiated cells were compared with mice given 3 immunizations at 0, 14 and 21 days, for their capacity to resist a challenge of $10^7$ unirradiated but $^{125}$IUdR-labelled BP8 on Day 28. Fig. 3 shows that repeated exposure to irradiated tumour does improve cytotoxic immunity as assessed by $^{125}$I clearance, over that seen when a single injection of irradiated cells was given. Also, 4/6 mice receiving 3 immunizations, rejected the tumour challenge, although 2 of them, who showed slower $^{125}$I loss, developed ascites and died on Days 14 and 21 respectively. As with the previous experiment, none of the mice receiving a single immunization with irradiated cells survived the live-tumour challenge.

### DISCUSSION

The use of $^{125}$IUdR in these experiments has allowed us to study the relative contributions of Cyclo and host immunity to the elimination of this potentially lethal ascites tumour. The wash-out studies in Cyclo-treated CBA mice showed that the recoverable tumour load 10 days after treatment was $\sim 10^5$ cells. However, Cyclo does delay the build up of tumour to the level of overt ascites by up to 2 weeks. It may be that this delay alone is sufficient to allow protective immunity to develop. However, the tentative conclusion which can be drawn from the experiments in which mice were immunized with irradiated cells is that Cyclo may actually facilitate the expression of immunity. The idea that this drug can produce such an effect is by no means new. Otterness and Chang (1976) showed an increase in T-cell cytotoxicity against a syngeneic tumour in mice after a single dose of 10 mg/kg of Cyclo, given at the same time as tumour cells. However, different timings and doses of drug reduced T-cell cytotoxicity. Askenase *et al.* (1975) showed an increase in delayed hypersensitivity reactions to sheep red cells when mice were tested 10 days after receiving 20 mg/kg of Cyclo. These mice had been given a primary immunization the day after the drug. Finerty and Krehl (1976) also showed potentiation of immunity in mice against plasmodium following Cyclo treatment. On the other hand, there are also many references in which Cyclo has been shown to be an active immunosuppressive agent (Berenbaum, 1975).

If Cyclo is potentiating immunity, a number of possible mechanisms come to mind. Firstly the potentiation may simply be due to the drug preserving antigen in a non-lethal form for many days within the animal. The increased immunity seen when 3 immunizations with heavily irradiated cells are used, compared with one, indicates that length of antigen exposure is an important factor, and this alone may provide an adequate explanation for the good immunity seen after Cyclo. It is also possible that the drugs may modify the antigenicity of the tumour cells to increase their immunogenicity. Such an effect has been recorded for tumour cells exposed to ionizing irradiation (Haddow and Alexander, 1964; Mathé *et al.*, 1969; Bomford, 1975; McBurney, 1976). Alternative mechanisms of potentiation include the modification of antigen processing or the inhibition of homoeostatic mechanisms (Ramshaw *et al.*, 1976).

CBA mice given a single i.p. immunization with heavily irradiated BP8 show clear evidence of weak cytotoxic immunity, as assessed by $^{125}$I loss, against a subsequent challenge of live BP8 cells (Table IV). However, these mice die significantly more rapidly from tumour than non-immunized mice do. This paradoxical result poses a number of interesting questions. Firstly does this finding mean that increased $^{125}$I loss in this instance is not reflecting true tumour-cell destruction? There are many reasons for delay in $^{125}$I loss from mice following destruction of $^{125}$IUdR-labelled tumour cells, including phagocytosis of dead cell debris, uptake of iodide by the thyroid, and impaired renal excretion. However, it is difficult to think of reasons for falsely high rates of $^{125}$I loss, other than injection of tumour into the gut. This gives a far more rapid loss of isotope than that seen in the immunized group of mice. In interpreting these data, one has to remember that BP8 is a rapidly dividing tumour. Mean cell-cycle times during the initial exponential growth phase after i.p. injection are around 12–14 h. Consequently, whilst 20% $^{125}$I excretion during the first day after the injection of $10^7$ $^{125}$IUdR-labelled BP8, reflects death of $2 \times 10^6$ cells, the same percentage loss 2 days later might reflect the death of $2 \times 10^7$ cells. The tumour-cell destruction rates in the mice immunized with a single injection of irradiated BP8

(Fig. 3) are never great enough to counter the exponential growth potential of BP8. Consequently, cure by this level of cytotoxic immunity would not be expected. Despite these arguments one still has to explain the more rapid death in mice immunized with irradiated cells than in controls. The way in which this tumour kills the host is not fully clear. In common with other experimental tumours, exponential growth does not continue indefinitely. Here the maximum number of tumour cells recoverable from the peritoneal cavity is between 5 and $8 \times 10^8$ cells, and before death this number often falls. It would seem reasonable to postulate that the rapid death in mice immunized in this way is related to alterations in the poorly understood events occurring when net tumour growth has effectively stopped. Without the use of $^{125}$IUdR studies the reduced survival time associated with immunization might have been attributed to blocking of cytotoxic immunity. These studies suggest that immunological enhancement in some instances may be attributable to quite different mechanisms.

We would like to thank Dr T. R. Munro for his work in establishing the $^{125}$IUdR technique in our laboratory. This work was supported by the Cancer Research Campaign, the Délégation Générale à la Recherche Scientifique et Technique and the Minnie Dawson Cancer Trust.

### REFERENCES

AMIEL, J. L. & BERARDET, M. (1974) Factor time for active immunotherapy after cytoreductive chemotherapy. *Eur. J. Cancer*, **10**, 89.

ASKENASE, P. W., HAYDEN, B. J. & GERSHON, R. K. (1975) Augmentation of delayed type hypersensitivity by doses of cyclophosphamide which do not affect antibody responses. *J. Exp. Med.*, **141**, 698.

BERENBAUM, M. C. (1975) The clinical pharmacology of immunosuppressive agents. In *Clinical Aspects of Immunology*. Eds. Gell, P. G. H., Coombs, R. R. A. and Lachman, P. J. Oxford: Blackwell. p. 689.

BOMFORD, R. (1975) Active specific immunotherapy of murine methylcholanthrene induced tumour with *C. parvum* and irradiated tumour cells. *Br. J. Cancer*, **32**, 551.

CHASSOUX, D. M., MACLENNAN, I. C. M. & MUNRO, T. R. (1977) Competition for cytotoxic immune capacity against a 'syngeneic' mouse tumour distributed at two sites. *Int. J. Cancer*, **19**, 796.

CURRIE, G. A. & BAGSHAWE, K. D. (1970) Active immunotherapy with *Corynebacterium parvum* and chemotherapy in murine fibrosarcomas. *Br. Med. J.*, *i*, 541.

FALCÃO, R. P., SONIS, S., MACLENNAN, I. C. M., CHASSOUX, D., DAVIES, A. J. S. & MUNRO, T. R. (1977) Assessment of drug sensitivity of human leukaemic myeloblasts: I Labelling human myeloblasts with $^{125}$IUdR for survival studies in mice. *Br. J. Cancer*, **36**, 297.

FINERTY, J. F. & KREHL, E. P. (1976) Cyclophosphamide pretreatment and protection against Malaria. *Infect. Immun.*, **14**, 1103.

FISHER, B., WOLMARK, N., RUBIN, H. & SAFFER, E. (1975) Further observations on the inhibition of tumour growth by *Corynebacterium parvum* with cyclophosphamide. I: Variation in administration of both agents. *J. Natl. Cancer Inst.*, **55**, 1147.

GOTOHDA, E., SENDO, F., HOSOKAWA, M., KODAMA, T. & KOBAYASHI, H. (1974) Combination of active and passive immunisation and chemotherapy to transplantation of methylcholanthrene-induced tumour in WKA rats. *Cancer Res.*, **34**, 1947.

HADDOW, A. & ALEXANDER, P. (1964) An immunological method of increasing the sensitivity of primary sarcomas to local irradiation with x-rays. *Lancet*, **i**, 452.

HOFER, K. G., PRENSKY, W. & HUGHES, W. L. (1970) Death and metastatic distribution of tumour cells in mice monitored with $^{125}$I iododeoxyuridine. *J. Natl. Cancer Inst.*, **43**, 763.

HUGHES, W. L., COMMERFORD, S. L., GITLIN, D., KONEGER, R. C., SCHULTZE, B., SHAH, V. & REILLY, P. (1964) Deoxyribonucleic acid metabolism *in vivo*. I—Cell proliferation and death as measured by incorporation and elimination of iodo deoxyuridine. *Fed. Proc.*, **23**, 640.

MCBURNEY, J. P. (1976) The destruction of IUdR-labelled tumour cells by immune responses and gamma-radiation studied *in vivo*. *M.Sc. Thesis. University of Oxford.*

MATHÉ, G., POUILLART, P. & LAPEYRAQUE, F. (1969) Active immunotherapy of L.1210 leukaemia applied after the graft of tumour cells. *Br. J. Cancer*, **23**, 814.

MIHICH, E. (1969) Combined effects of chemotherapy and immunity against leukaemia L1210 in DBA/2 mice. *Cancer Res.*, **29**, 848.

MOORE, M. & WILLIAMS, D. E. (1973) Contribution of host immunity to cyclophosphamide therapy of a chemically induced murine sarcoma. *Int. J. Cancer*, **11**, 358.

OTTERNESS, I. G. & YI-HAN CHANG (1976) Comparative study of cyclophosphamide, 6-mercaptopurine, azathiopurine and methotrexate. Relative effects on the humoral and the cellular immune response in the mouse. *Clin. Exp. Immunol.*, **26**, 346.

PEARSON, J. W., CHIRIGOS, M. A., CHAPAPAS, S. D. & SHER, N. A. (1974) Combined drug and immunostimulation therapy against a syngeneic murine leukaemia. *J. Natl. Cancer Inst.*, **52**, 463.

PORTEOUS, D. D. & MUNRO, T. D. (1972) The kinetics of the killing of mouse tumour cells *in vivo* by immune responses. *Int. J. Cancer*, **10**, 112.

RAMSHAW, I. A., BRETSCHER, P. A. & PARISH, C. R. (1976) Regulation of the immune response. I: Suppression of delayed-type hypersensitivity by T cells from mice expressing humoral immunity. *Eur. J. Immunol.*, **6**, 674.